# Falls in Older Adults Requiring Emergency Services: Mortality, Use of Healthcare Resources, and Prognostication to One Year

**DOI:** 10.5811/westjem.2021.11.54327

**Published:** 2022-05-14

**Authors:** Craig D. Newgard, Amber Lin, Aaron B. Caughey, K. John McConnell, Eileen Bulger, Susan Malveau, Kristan Staudenmayer, Denies Griffiths, Elizabeth Eckstrom

**Affiliations:** * Oregon Health & Science University, Center for Policy and Research in Emergency Medicine, Department of Emergency Medicine, Portland, Oregon; † Oregon Health & Science University, Department of Obstetrics and Gynecology, Portland, Oregon; ‡ Oregon Health & Science University, Center for Health Systems Effectiveness, Department of Emergency Medicine, Portland, Oregon; § University of Washington, Department of Surgery, Seattle, Washington; ¶ Stanford University Medical Center, Department of Surgery, Palo Alto, California; || Oregon Health & Science University, Division of General Internal Medicine & Geriatrics, Portland, Oregon

## Abstract

**Introduction:**

Older adults who fall commonly require emergency services, but research on long-term outcomes and prognostication is sparse. We evaluated older adults transported by ambulance after a fall in the Northwestern United States (US) and longitudinally tracked subsequent healthcare use, transitions to skilled nursing, hospice, mortality, and prognostication to one year.

**Methods:**

This was a planned secondary analysis of a cohort study of community-dwelling older adults enrolled from January 1–December 31, 2011, with follow-up through December 31, 2012. We included all adults ≥ 65 years transported by 44 emergency medical services agencies in seven Northwest counties to 51 hospitals after a fall. We matched Medicare claims, state inpatient data, state trauma registry data, and death records. Outcomes included mortality, healthcare use, and new claims for skilled nursing and hospice to one year.

**Results:**

There were 3,159 older adults, with 147 (4.7%) deaths within 30 days and 665 (21.1%) deaths within one year. There was an initial spike in inpatient days, followed by increases in skilled nursing and hospice. We identified four predictors of mortality: respiratory diagnosis; serious brain injury; baseline disability; and Charlson Comorbidity Index ≥ 2. Having any of these predictors was 96.6% sensitive (95% confidence interval [CI]: 95.7, 97.5%) and 21.4% specific (95% CI: 19.9, 22.9%) for 30-day mortality, and 91.6% sensitive (95% CI: 89.5, 93.8%). and 23.8% specific (95% CI: 22.1, 25.5%) for one-year mortality.

**Conclusion:**

Community-dwelling older adults requiring ambulance transport after a fall have marked increases in healthcare use, institutionalized living, and mortality over the subsequent year. Most deaths occur following the acute care period and can be identified with high sensitivity at the time of the index visit, yet with low specificity.

## INTRODUCTION

In the United States 25% of older adults fall each year,^1^,^2^ a portion of whom require ambulance transport to an emergency department (ED). Whether a fall requiring an ambulance represents a marker of declining health (eg, due to increasing comorbidity burden, cognitive impairment, or progressive physical limitations) or a sentinel event indicating a more rapid downward trajectory (possibly from the fall itself) remains unclear. There is a growing body of research and guidelines on fall prevention,^3^,^4^ yet prognostication and the practical implications after a fall are not well understood. Guidance on these topics using real-world data would be useful to acute care physicians, primary care clinicians, patients, and families.

Among older adults injured by a variety of mechanisms and requiring ambulance transport, a fall mechanism was independently associated with death within one year.^5^ Other research has described the management and outcomes of older adults incurring a hip fracture from a fall.^6^ However, the effect of a fall on subsequent healthcare use, long-term mortality, and methods for risk-stratifying patients remain poorly described. We hypothesized that a fall requiring ambulance transport represents a sentinel event among older adults, portending increased need for healthcare resources, transitions in living environment, and high mortality. We also hypothesized that information available at the time of the index visit could be used to identify patients with high short- and long-term mortality risk.

In this study we analyzed a cohort of community-dwelling older adults requiring ambulance transport after a fall to evaluate subsequent healthcare use (ambulance transports, ED visits, inpatient days, skilled nursing days, and hospice), mortality, and mortality prognostication using information available during the index ED/hospital visit. We sought to generate practical information to guide clinicians, patients, and families about what to expect in the year after a fall requiring ambulance transport.

## METHODS

### Study Design

This was a planned secondary analysis of a retrospective cohort study^5^ reviewed and approved by institutional review boards in all study sites, with waiver of the requirement for informed consent. We used the Strengthening the Reporting of Observational Studies in Epidemiology (STROBE) cohort study guidelines.^7^

### Study Setting

We conducted the study in seven counties in the Northwestern US, including two major metropolitan areas Portland, Oregon and Seattle, Washington and two rural counties. Forty-four emergency medical services (EMS) agencies serve these counties and transport to 51 acute care hospitals. We included six additional hospitals after tracking interhospital transfers from the initial receiving facility. The 57 hospitals have varying capabilities and services, and included the following: three Level I trauma centers; seven Level II trauma centers; 10 Level III trauma hospitals; nine Level IV hospitals; one Level V hospital; and 27 non-trauma hospitals. In US trauma systems, Level I hospitals are equipped to care for the most complex trauma patients. Level II, III, and IV hospitals each have sequentially fewer comprehensive resources and capacity to care for trauma patients.

**Population Health Research Capsule**

What do we already know about this issue?

*Although falls among older adults are common and frequently require emergency services, research on long-term outcomes and prognostication is sparse.*

What was the research question?

*Among older adults who fall, what is healthcare use and mortality to one year? Can mortality be predicted during the index visit?*

What was the major finding of the study?

*There was a large increase in healthcare services and death after a fall and mortality can be predicted during the index visit.*

How does this improve population health?

*These findings provide practical information to guide clinicians, patients, and families about expectations in the year after a fall requiring ambulance transport.*

### Patient Population

We included consecutive, community-dwelling adults ≥ 65 years with Medicare fee-for-service coverage with a fall requiring ambulance transport to an acute care hospital from January 1–December 31, 2011, with follow-up through December 31, 2012. We required that patients had continuous Medicare fee-for-service coverage for one year before and after transport (or until death) to provide comprehensive information about baseline function, comorbidities, frailty, and healthcare utilization. Medicare patients without fee-for-service coverage (ie, Medicare Advantage) function under a different payment model that generates different claims data, which are less useful for research. We included patients regardless of the receiving hospital, their injury severity, or admission status. We restricted the sample to community-dwelling older adults to minimize the effect of patients with different goals of care (eg, institutionalized patients). We excluded patients with any of the following in the year prior to ambulance transport: skilled nursing facility (SNF) claim; hospice claim; or Provider Order for Life Sustaining Treatment (POLST) form specifying “limited interventions” or “comfort measures only” (Oregon only).

### Data Processing

We collected EMS data as part of a prospective, all-age cohort study evaluating field trauma-triage processes in the seven counties.^8^ We then used probabilistic linkage^9^ (LinkSolv, v.9.0.0190, Strategic Matching, Inc., Morrisonville, NY) to match the EMS data to state trauma registries (two), state hospital discharge databases (two), state death certificate data (two), and the Oregon electronic POLST registry. An external contractor for the Centers for Medicare and Medicaid Services deterministically matched the EMS data to Medicare claims data for one year before and after the date of 9-1-1 contact. We have validated the electronic data processing methods used in the study, including probabilistic linkage, multiple imputation, and development of key variables.^10^

### Variables

We included the following variables: age; gender; field trauma-triage status; initial prehospital physiologic measures (Glasgow Coma Scale score, systolic blood pressure, respiratory rate, and heart rate); prehospital procedures; mode of transport (ground vs air); and initial receiving hospital. We calculated baseline health measures using Medicare claims and other record sources available for the year prior to ambulance transport, including the following: Charlson Comorbidity Index (CCI)^11^; individual comorbidities; modified Frailty Index;^12^ and a claims-based measure of functional disability.^13^,^14^ The claims-based disability measure was derived and validated in a Medicare population and represents the probability (from 0 to 1) of serious disability, with a threshold of 0.11 indicating limited self-care (confined to a bed or chair more than 50% of waking hours), or completely disabled.^13^,^14^ Finally, we quantified baseline healthcare use for the year prior to transport (ambulance transports, ED visits, and inpatient days).

We considered the initial ED visit associated with ambulance transport after the fall to be the “index” visit, whether or not a patient required admission. Variables from the index visit included the following: Abbreviated Injury Scale (AIS) score for different body regions;^15^ Injury Severity Score (ISS)^15^,^16^; *International Classification of Diseases, 9^th^ Rev, Clinical Modification* (ICD-9-CM) diagnosis codes; ICD-9-CM procedure codes, categorized using the Agency for Healthcare Research and Quality Clinical Classification System (CCS);^17^ and interhospital transfer. Because AIS and ISS are not available in administrative data sources, we used a mapping function, the ICDPIC module for Stata v11 (StataCorp, College Station, TX) to convert ICD-9-CM diagnosis codes into injury severity measures,^17^ which has been validated.^18^ We also mapped ICD-9-CM diagnosis codes from the index visit to characterize common fracture patterns among older adults, including the following: femoral neck fractures; other femur fractures; pelvic fractures; tibia and fibula fractures; humerus fractures; and radius or ulna fractures. For hospital procedures, we combined CCS categories into major non-orthopedic surgery (brain, spine, neck, chest, and abdominal-pelvic operations), orthopedic surgery, and blood transfusion.

### Outcomes

The primary outcomes were mortality and healthcare use from the date of ambulance transport to one year. We measured the following types of healthcare use: inpatient days; ambulance transports; ED visits; SNF days; and hospice days. Among patients who died within one year, we evaluated causes of death (including the primary cause and contributing factors) using ICD-9-CM diagnosis codes from matched death certificates and the location of death.

### Data Analysis

We used descriptive statistics to characterize patients, diagnoses, procedures, healthcare use, and mortality. The percent missingness for variables ranged from 0–28.4%, with most variables having less than 4% missing values and mortality with 0% missing (Supplemental eTable 1). To handle missing values and minimize bias, we used multiple imputation.^19^ We generated 10 multiple imputed datasets using flexible chains regression models^21^ (IVEware v0.1, University of Michigan, MI), and then combined the results using Rubin’s rules to account for overall variance.^19^

To derive a prognostication tool for short-term (30-day) and long-term (one-year) mortality, we used classification and regression tree (CART) analysis, v8.0^22^ (Salford Systems, San Diego, CA). The CART analysis is a non-parametric method of binary recursive partitioning^23^ well suited for the development of clinical decision rules that classifies observations based on many possible predictor variables, including the identification of higher level interactions.^22^ It also allows for data-driven selection of cut-points for continuous variables, rather than reliance on pre-selected, arbitrary values. We selected misclassification costs and Gini splitting functions^22^ to derive a decision rule with a sensitivity ≥ 95%. The CART analysis uses the “cost-complexity” method for pruning decision trees, which prunes terminal nodes (lower branches) if the additional accuracy gained by the branch is minimal in comparison to tree complexity.^22^ We selected parameters for tree complexity that facilitated development of a practical and sensible decision tree that would be feasible for clinical use.

To reduce the potential for overfitting the dataset and to minimize bias, we used 10-fold cross-validation methods to select the final decision tree.^24^,^25^ Cross validation is a process that uses approximately 90% of the sample to derive the rule and 10% of the sample for validation, and then replicates this process until every patient has been used at least once to both derive and validate the decision tool. The CART analysis included 51 predictor variables with a goal of identifying 95% of patients dying within 30 days and (separately) within one year. We included all available predictors with known or plausible a priori association with mortality that could reasonably be known and available to clinicians and families during the index visit: patient demographics; healthcare use over the prior year; baseline function (disability status and frailty); comorbidities (CCI, total comorbidity count, and 13 individual comorbidities); hospital procedures; and diagnoses (14 categories). We analyzed each of the 10 multiple imputed datasets independently and then combined the results into a final decision tree.

As a complement to CART analysis, we also used a multivariable logistic regression model with the same variables to assess factors independently associated with 30-day and one-year mortality. We removed variables with multicollinearity and calculated area under the receiver operating characteristic (AUROC) curve to determine model discrimination. We adjusted all measures of variance for multiple imputation. We examined model diagnostics to assess goodness of fit, influential values, and multicollinearity. We used SAS v9.4 (SAS Institute, Cary, NC) for these analyses.

## RESULTS

**Figure 1:**
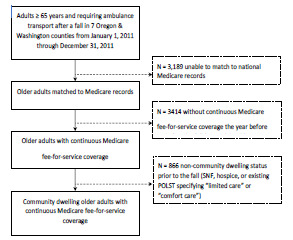
Schematic of cohort creation.

Of the 10,628 older adults transported by ambulance after a fall, 4,025 had matched Medicare fee-for-service records for one year before and after 9-1-1 contact, and 3,159 met our criteria for community-dwelling older adults (Figure 1). Comparison of patients who were included (N = 3,159) vs excluded (N = 7,469) demonstrated similar demographics, initial physiologic measures, selection of receiving hospitals, and one-year mortality (Supplemental eTable 2).

Of the 3,159 patients in the primary sample, 147 (4.7%) patients died within 30 days and 665 (21.1%) died within one year. The sample was 70% female, with a median age of 84 years (interquartile range [IQR] 77–89), and 84% had at least one comorbidity. Baseline disability was low, as was use of healthcare services for the year prior to the fall event. During the index visit, non-injury diagnoses were common. There were 173 (5.5%) patients with overall serious injury (ISS ≥ 16). Serious extremity injury was the most common type of injury (N = 593, 18.8%), and orthopedic surgery was the most common hospital intervention (N = 571, 18.1%). We characterize the sample in Table 1.

**Figure 2:**
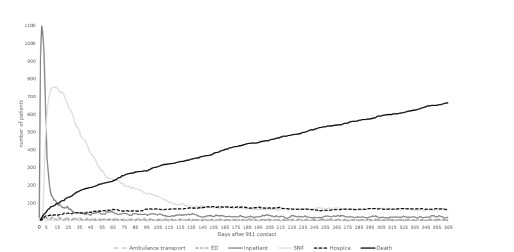
Changes in healthcare use and mortality to one year among community-dwelling older adults requiring ambulance transport after a fall (N = 3159). *ED*, emergency department; *SNF*, skilled nursing facility. *Initial ambulance transports and ED visits are not illustrated here, as every patient in the sample was transported to an ED by ambulance on day zero.

In Figure 2 we show daily use of healthcare resources after ambulance transport and mortality to one year. There was a sharp initial spike in inpatient days following transport, reflecting the 1404 (44.4%) patients requiring admission during the index visit. However, the number of patients remaining inpatient declined quickly (median length of hospital stay three days, IQR 0–7 days), concurrent with a rise in use of skilled nursing facilities within two days of transport. Use of skilled nursing peaked at 10 days and then slowly declined to a relatively stable rate by 140 days. Use of hospice also rose shortly after transport, plateauing at 140 days. Mortality rose quickly in the first two weeks, and then followed a linear upward slope over the subsequent year, without a plateau.

**Figure 3:**
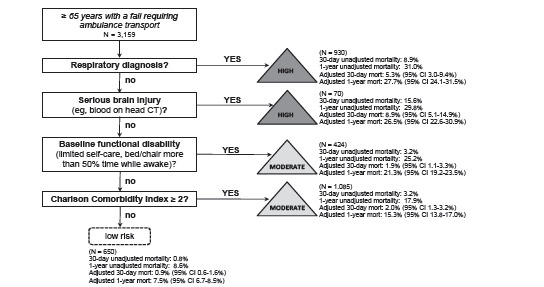
Prognostication tool predicting 30-day and one-year mortality using information from the index visit among community-dwelling older adults requiring ambulance transport after a fall. *CT*, computed tomography; *CI*, confidence interval; *mort*, mortality.

We illustrate the prognostication tool in Figure 3 and accuracy measures in Table 2. We identified four predictors of mortality available at the time of the index visit (in order): a respiratory diagnosis; serious brain injury (head AIS ≥ 3); baseline disability; and CCI ≥ 2. The prognostication tool (any of the four predictors) had sensitivity of 96.6% (95% confidence interval [CI]: 95.7, 97.5%) and specificity of 21.4% (95% CI: 19.9, 22.9%) for identifying patients dying within 30 days of ambulance transport (AUROC 0.69). For prediction of patients dying within one year, the tool had sensitivity of 91.6% (95% CI: 89.4, 93.8%) and specificity of 23.8% (95% CI: 22.1, 25.5%) (AUROC 0.64). Crude and adjusted one-year mortality decreased in each step of the prognostication tool, with some fluctuation in 30-day mortality (crude and adjusted) across the steps. Patients not meeting the four criteria had 0.8% 30-day mortality (adjusted 30-day mortality 0.9%) and 8.6% one-year mortality (adjusted one-year mortality 7.5%). Among the 705 patients with a respiratory diagnosis from the index visit, there were 201 unique combinations of 1306 respiratory diagnoses. Chronic obstructive pulmonary disease was the most common (147 of 705 patients, 20.9%; 279 of 1306 respiratory diagnoses, 21.4%) (eTable 3).

Results from multivariable models for 30-day and one-year mortality are shown in Table 3. Respiratory diagnosis, head injury, age, and mechanical ventilation were all independent predictors of 30-day and one-year mortality. Additional predictors of mortality at both time points included cancer diagnosis, dementia diagnosis, and hip fracture, while orthopedic surgery and female gender had protective effects at both time points. Model discrimination was good for 30-day mortality (AUROC 0.83) and fair for one-year mortality (AUROC 0.75), with model diagnostics indicating a good model fit. We analyzed additional models using four common respiratory diagnosis subcategories (acute respiratory failure, pulmonary infections, chronic respiratory conditions, and other), which showed that acute respiratory failure was associated with 30-day mortality (but not one-year mortality), while the three other respiratory categories were associated with one-year mortality (but not 30-day mortality).

**Figure 4:**
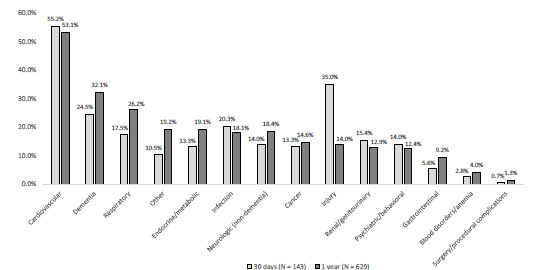
Causes of death at 30 days and one year among older adults who died after a fall (N = 629 patients with death certificate information). *Due to potential variability in the completion of death certificates, we considered all causes (primary and contributing causes) for these categories. Therefore, the categories are not mutually exclusive and do not add to 100%, but are comprehensive in detailing factors contributing to death.

Among the 665 patients who died within one year, 629 (95%) had death certificate information available. Cardiovascular causes were the most common cause of death at 30 days and overall (55% of deaths within 30 days and 53% of deaths overall) (Figure 4). Most other causes were similar at both time points, except for injury causes. Among patients dying within 30 days, 35% had an injury cause, yet injury was an uncommon cause of death overall (14%). The location of death was as follows: 40% skilled nursing or other long-term care facility; 23% ED/inpatient; 15% home; 12% residential care (including assisted living and adult foster care); 9% hospice; and 1% other.

## DISCUSSION

In this study we demonstrate marked changes in healthcare use, institutionalized living, and mortality among community-dwelling older adults requiring ambulance transport after a fall. The findings suggest that a fall requiring emergency services is a life-changing event for older adults, rather than simply a marker of steady decline. We also demonstrate that 30-day and one-year mortality for these patients can be predicted with high sensitivity using information available during the index visit. Prognostication after a fall event may have an important role in decision-making among clinicians, patients, and families. The four-variable decision tool had high sensitivity with relatively low specificity, which influences how this information might be used in practice. Because most patients who died had one of the four predictors, patients lacking these factors were much more likely to be alive at 30 days and one year after the event. However, based on the low specificity of the tool, having one of the four predictors did not necessarily put a patient at high risk of death; so this rule should not be used to guide decision-making about limiting or withdrawing care. Furthermore, the decision tool requires prospective validation before consideration for clinical use.

There were substantial and measurable increases in healthcare use, institutionalized care, hospice, and mortality after a fall requiring ambulance transport. While approximately one quarter of older adults fall each year,^1^,^2^ only a portion of these patients seek medical care.^26^ Because all patients in our cohort required ambulance transport, the sample represented a higher acuity population yet a familiar one to most clinicians and families in the US. Compared to baseline healthcare use and independent living, this event signaled a marked change for many patients. The findings show that in addition to the fall itself, concurrent respiratory conditions, head injury, baseline disability, and comorbidity burden are major factors changing the life trajectory of these patients. These results support ongoing efforts to prevent falls among older adults and provide insight about what to expect in the year after a fall. The prominent use of skilled nursing facilities for post-acute care in our sample was similar to research showing an increase in post-discharge use of these facilities and reduction in length of hospital stays among Medicare beneficiaries over time.^27^

Mortality rose quickly after transport and then followed a steady upward linear slope to one year. While the mortality curve was steepest in the first two weeks, most deaths occurred after the acute phase of care. Mortality did not plateau at any point in the subsequent year. The findings demonstrate the importance of risk-prediction beyond hospitalization. While injuries are often the clinical focus among older adults who fall, our results demonstrate the importance of non-injury conditions. Previous research has shown that brain injury^28^ and hip fracture^6^ are important causes of death among older adults who fall, which is supported by our findings. However, we also demonstrate the importance of respiratory conditions, baseline disability, and comorbidity burden. Among respiratory conditions identified during the index visit, adjusted mortality differed by the type of respiratory condition and when mortality was measured. Acute respiratory failure was predictive of short-term but not long-term mortality. Other respiratory conditions (acute respiratory infections, chronic pulmonary conditions, and other) had the opposite pattern, predicting long-term but not short-term mortality.

Methods to quantify fall-risk among older adults have been established,^3^,^4^ but we are not aware of prediction tools to quantify short- and long-term mortality after a fall. Multiple instruments have been developed to predict short-term mortality among older adults requiring hospital admission,^29^ but are not specific to older adults who fall or to the ED population. A previous study combined 18 predictor instruments into a composite prognostication tool for admitted older adults (Criteria for Screening and Triaging to Appropriate Alternative care, CriSTAL),^29^ which was subsequently validated in other countries.^30^,^31^ During the validation process for CriSTAL, the variables most predictive of short-term mortality included frailty, older age, male gender, advanced malignancy, nursing home residence, and low oxygen saturation.^30^,^31^

Our prognostication tool differed in that it focused on community-dwelling older adults who fell and required ambulance transport (whether or not the patient was admitted), had higher sensitivity and lower specificity for predicting mortality compared to CriSTAL, and included certain measures not identified for CriSTAL (serious brain injury and comorbidity burden). Similarities between our tool and CriSTAL included respiratory function and functional status. Other prediction tools for older adults have identified comorbidity burden, frailty, age, and cancer as important factors,^29^ which were similar in our multivariable model. While prognostication tools will differ in their ability to predict outcomes across different populations and different types of patients, focus on a specific type of patient (eg, older adults who fall) may improve accuracy, utility, and targeted decision-making. The high prevalence of falls among older adults in the US and the need for guidance in helping families and clinicians make early decisions suggest that tools are needed to quantify what to expect in the subsequent year and could be a useful complement to fall prevention efforts.

## LIMITATIONS

There were limitations in our study. The cohort was enrolled 10 years ago. While the cohort was unique in its development and capture of long-term outcomes, it is possible that patients and/or clinical care have changed during the interim period. Using publicly available, national non-fatal and fatal injury data from 2011 (study year) through 2018 (most recent available data) for older adults suggests that the population-based rate of non-fatal ED visits for falls among patients ≥ 65 years in the US did not substantially change,^32^ but that age-adjusted mortality rates have increased.^32^,^33^ While there have been national programs implemented during this time to prevent falls (eg, the Stopping Elderly Accidents, Deaths & Injuries program^4^), we are unaware of widespread changes in the clinical care of older adults after a fall. We limited the sample to patients with matched Medicare fee-for-service records, which was necessary to evaluate baseline healthcare use, comorbidities, frailty, and functional status. However, restricting the sample in this way eliminated the population-based sampling used for the original cohort and may have introduced selection bias.

Comparison of patients included vs excluded from the sample demonstrated similar demographics, initial physiology, ambulance transport patterns, and one-year mortality. In addition, we used the mechanism of injury recorded by EMS at the time of the event, but the fall mechanism was not separated by ground-level, fall from height (eg, a ladder), or fall down stairs, and did not detail the type of landing surface. The sample was drawn largely from two metropolitan areas in the Northwestern US. It is possible that older adults in other regions or countries with differing demographics, baseline disability, or comorbidity burden may have different trajectories and prognostication after a fall. Finally, other analytic approaches (eg, machine learning) may be able to derive a tool with higher predictive performance and prospective validation of our prognosti-cation tool will be important before these results are considered for clinical care.

## CONCLUSION

Our results show that a fall requiring ambulance transport represents a major shift in the lives of many older adults, with increased use of healthcare services, need for institutionalized living, hospice, and high mortality in the following year. We also demonstrate that patients dying within 30 days and one year can be identified with high sensitivity using information available during the index visit, but with low specificity, which affects how such information might be used in practice.

An abstract of the findings of this research was published for the Society for Academic Emergency Medicine Annual Meeting (May 2020).

## Supplementary Information



## Figures and Tables

**Table 1:** Characteristics of community-dwelling older adults requiring ambulance transport after a fall (N = 3,159).

Demographics		
Age in years – median (IQR)	84	(77–89)
65 – 74 years	591	(18.7%)
75 – 84 years	1,045	(33.1%)
85 – 94 years	1,323	(41.9%)
≥ 95 years	200	(6.3%)
Women	2,219	(70.3%)
Non-White race	189	(6.0%)
Pre-injury measures		
Comorbidities		
Charleston Comorbidity Index – median (IQR)	3	(1–5)
Myocardial infarction	1,084	(34.3%)
Dementia	1,000	(31.7%)
Congestive heart failure	897	(28.4%)
Renal insufficiency	841	(26.6%)
Diabetes	830	(26.3%)
COPD	819	(25.9%)
Cerebrovascular disease	782	(24.8%)
Cancer	626	(19.8%)
Peripheral vascular disease	622	(19.7%)
Rheumatoid arthritis	172	(5.4%)
Ulcers	59	(1.9%)
Paralysis	49	(1.6%)
Liver disease	33	(1.0%)
Modified frailty index – median (IQR)	2	(1–4)
Disability scale – median (IQR)	0.03	(0.01–0.08)
Ambulance transports over prior year – median (IQR)	0	(0–1)
ED visits over prior year – median (IQR)	0	(0–1)
Inpatient days over prior year – median (IQR)	0	(0–0)
Initial (prehospital) physiology		
GCS ≤ 8	13	(0.4%)
GCS 9 – 12	71	(2.2%)
GCS 13 – 15	3,075	(97.4%)
SBP ≤ 100 mm Hg	144	(4.6%)
Type of initial receiving hospital		
Level I	216	(6.8%)
Level II	234	(7.4%)
Level III–V	1,326	(42.0%)
Non-trauma hospital	1,383	(43.8%)
Index ED/Hospital visit		
Diagnosis categories^a^		
Injury	3,033	(96.0%)
Cardiovascular	2,350	(74.4%)
Endocrine/metabolic	1,516	(48.0%)
Neurologic (non-dementia)	1,145	(36.3%)
Respiratory	930	(29.4%)
Psychiatric/behavioral	922	(29.2%)
Gastrointestinal	740	(23.4%)
Blood disorders/anemia	734	(23.2%)
Renal/genitourinary	698	(22.1%)
Infection	688	(21.8%)
Surgical/procedural complication	464	(14.7%)
Dementia	327	(10.4%)
Cancer	237	(7.5%)
Injury patterns		
Minor, ISS 0 – 8	1,828	(57.9%)
Moderate, ISS 9 – 15	1,158	(36.7%)
Serious, ISS 16 – 24	156	(4.9%)
Severe, ISS ≥ 25	17	(0.5%)
Serious head injury, AIS ≥ 3	148	(4.7%)
Serious chest injury, AIS ≥ 3	40	(1.3%)
Serious abdominal-pelvic injury, AIS ≥ 3	5	(0.2%)
Serious extremity injury, AIS ≥ 3	593	(18.8%)
Fracture patterns		
Femoral neck	519	(16.4%)
Other femur	120	(3.8%)
Pelvis	142	(4.5%)
Tibia and/or fibula	104	(3.3%)
Humerus	181	(5.7%)
Radius and/or ulna	134	(4.3%)
Hospital interventions		
Major non-orthopedic surgery	54	(1.7%)
Orthopedic surgery	571	(18.1%)
Blood transfusion	245	(7.8%)
Intubation/mechanical ventilation^b^	47	(1.5%)
Interhospital transfer	277	(8.8%)
Outcomes to one year		
Mortality		
7-day mortality	63	(2.0%)
30-day mortality	147	(4.7%)
90-day mortality	277	(8.8%)
365-day mortality	665	(21.1%)
Resource use		
Post-index ambulance transports^b^ – median (IQR)	0	(0–1)
Post-index ED visits^b^ – median (IQR)	1	(0–2)
Inpatient days, including index visit – median (IQR)	3	(0–7)
Post-index SNF days - median (IQR)	0	(0–23)
Hospice claim	421	(13.3%)

*IQR*, interquartile range; *SBP*, systolic blood pressure; *mm Hg*, millimeters of mercury; *GCS*, Glasgow Coma Scale score; *ISS*, Injury Severity Score; *AIS*, Abbreviated Injury Scale score; *COPD*, chronic obstructive pulmonary disease.

a Patients could have multiple diagnoses; therefore, percentages do not add to 100%.

b Intubation/mechanical ventilation includes patients arriving intubated to the emergency department (ED) (prehospital intubation). Ambulance transports and ED visits do not include the index event, as all patients in the cohort were transported by ambulance to the ED following 9-1-1 contact.

**Table 2:** Prediction accuracy for short- and long-term mortality among older adults who fell and required ambulance transport (N = 3,159).

	30-day mortality		One-year mortality	
Sensitivity	96.6%	(95% CI: 95.7, 97.5%)	91.6%	(95% CI: 89.4, 93.8%)
Specificity	21.4%	(95% CI: 19.9, 22.9%)	23.8%	(95% CI: 22.1, 25.5%)
Positive predictive value	5.7%	(95% CI: 1.9, 9.4%)	24.3%	(95% CI: 21.0, 27.6%)
Negative predictive value	99.2%	(95% CI: 99.1, 99.3%)	91.5%	(95% CI: 90.3, 92.6%)
Likelihood ratio +	1.23	(95% CI: 1.18, 1.27)	1.20	(95% CI: 1.16, 1.24)
Likelihood ratio −	0.16	(95% CI: 0.02, 0.30)	0.35	(95% C:I 0.26, 0.44)

**Table 3:** Multivariable model for 30-day and one-year mortality among community-dwelling older adults transported by ambulance after a fall (N = 3,159).

Variable	Odds ratio of 30-day mortality (95% CI)	*P*-value	Odd ratio of one-year mortality (95% CI)	*P*-value
Pre-index EMS use over past 1 year	0.80 (0.60–1.07)	0.13	0.97 (0.88–1.08)	0.61
Pre-index ED visits over past 1 year	1.06 (0.89–1.25)	0.53	1.02 (0.94–1.10)	0.65
Pre-index inpatient days over past 1 year	1.00 (0.96–1.04)	0.89	1.03 (1.01–1.05)	0.01
Age	1.06 (1.03–1.08)	<0.01	1.06 (1.05–1.07)	<0.01
Female	0.54 (0.36–0.79)	<0.01	0.49 (0.40–0.60)	<0.01
Total comorbidity count	1.08 (0.91–1.29)	0.35	1.11 (1.01–1.21)	0.02
Baseline disability score	1.92 (0.63–5.89)	0.25	2.47 (1.43–4.27)	<0.01
Modified frailty index	0.97 (0.80–1.17)	0.75	1.02 (0.93–1.12)	0.69
Head injury severity (head AIS)	4.07 (2.35–7.06)	<0.01	1.69 (1.13–2.53)	0.01
Chest injury severity (chest AIS)	0.60 (0.14–2.54)	0.49	0.97 (0.44–2.14)	0.93
Extremity injury severity (extremity AIS)	1.83 (0.59–5.63)	0.29	1.32 (0.62–2.83)	0.47
Fracture – pelvis	0.69 (0.29–1.61)	0.39	0.69 (0.43–1.12)	0.14
Fracture – hip	4.44 (1.4–14.02)	0.01	2.29 (1.04–5.04)	0.04
Fracture – femur (non-hip)	1.70 (0.72–4.01)	0.23	0.89 (0.51–1.54)	0.67
Fracture – tibia	1.36 (0.31–5.97)	0.69	0.82 (0.39–1.72)	0.59
Fracture – humerus	2.79 (1.43–5.46)	<0.01	0.94 (0.60–1.48)	0.81
Fracture – radius	1.35 (0.52–3.52)	0.54	0.94 (0.55–1.61)	0.82
Orthopedic surgery during index event	0.14 (0.06–0.31)	<0.01	0.33 (0.19–0.56)	<0.01
Non-orthopedic surgery during index event	3.44 (1.44–8.20)	0.01	1.44 (0.70–2.97)	0.33
Need for intubation/mechanical ventilation	7.68 (3.52–16.74)	<0.01	2.28 (1.17–4.45)	0.02
Blood transfusion during index event	0.68 (0.32–1.44)	0.31	1.31 (0.89–1.93)	0.18
Inter-hospital transfer	1.09 (0.60–1.99)	0.78	1.09 (0.76–1.56)	0.63
Index event – blood/anemia diagnosis	0.96 (0.60–1.52)	0.85	1.07 (0.82–1.39)	0.62
Index event – cancer diagnosis	1.87 (1.08–3.22)	0.03	1.87 (1.35–2.59)	<0.01
Index event – cardiovascular	0.93 (0.52–1.65)	0.79	0.76 (0.58–0.98)	0.03
Index event – dementia diagnosis	1.92 (1.19–3.10)	0.01	1.64 (1.24–2.17)	<0.01
Index event – endocrine	0.98 (0.64–1.50)	0.94	1.08 (0.87–1.35)	0.48
Index event – gastrointestinal	1.14 (0.75–1.74)	0.53	0.78 (0.61–1.00)	0.05
Index event – infection diagnosis	1.62 (1.07–2.44)	0.02	1.21 (0.96–1.54)	0.11
Index event – injury diagnosis	0.67 (0.27–1.65)	0.38	0.80 (0.48–1.33)	0.38
Index event – neurologic diagnosis (non-dementia)	1.20 (0.79–1.81)	0.40	1.15 (0.93–1.41)	0.20
Index event – other	1.25 (0.62–2.53)	0.54	1.23 (0.90–1.68)	0.19
Index event – psychiatric/behavioral diagnosis	0.94 (0.62–1.42)	0.77	1.19 (0.95–1.48)	0.13
Index event – renal diagnosis	1.21 (0.79–1.84)	0.38	1.19 (0.93–1.52)	0.16
Index event – respiratory diagnosis	2.24 (1.47–3.41)	<0.01	1.87 (1.50–2.34)	<0.01
Index event – surgical complication diagnosis	0.69 (0.37–1.30)	0.26	0.64 (0.45–0.90)	0.01
c-statistic	0.824		0.754	

*CI*, confidence interval; *EMS*, emergency medical services; *ED*, emergency department; *AIS*, Abbreviated Injury Scale.

Variables excluded from the model due to collinearity included: individual comorbidities, Charlson Comorbidity Index, and abdominal-pelvis Abbreviated Injury Scale score.
