# Supplementary material for: Falls in Older Adults Requiring Emergency Services: Mortality, Use of Healthcare Resources, and Prognostication to One Year
Source: West J Emerg Med. 2022 May 14;23(3):375–85. doi: 10.5811/westjem.2021.11.54327 (PMC9183773; doi:10.5811/westjem.2021.11.54327)
Supplement: Supplementary file 1 [file wjem-23-375-s001.docx]

**Supplemental On-Line Materials.**

**eTable 1.** Missingness for variables among older adults transported by ambulance after a fall

(n = 3,159).

|  | **% missingness** |
| --- | --- |
| Age | 0.0% |
| Sex | 1.6% |
| Charleston Comorbidity Index | 0.0% |
| Modified frailty index | 0.0% |
| Ambulance transports over prior year | 0.0% |
| ED visits over prior year | 0.0% |
| Inpatient days over prior year | 0.0% |
| EMS initial GCS | 28.4% |
| EMS initial SBP | 7.0% |
| EMS initial Heart rate | 6.8% |
| EMS initial Respiratory rate | 11.5% |
| Type of initial receiving hospital: | 0.0% |
| Index ED/hospital diagnosis categories | 3.5% |
| Injury Severity Score (ISS) | 3.5% |
| Abbreviated Injury Scale (AIS) scores | 3.5% |
| Fracture patterns (based on ICD9 diagnosis codes) | 3.5% |
| Major non-orthopedic surgery | 3.5% |
| Orthopedic surgery | 3.5% |
| Blood transfusion | 3.5% |
| Intubation/mechanical ventilation | 3.5% |
| Inter-hospital transfer | 5.9% |
| Ambulance transports ^-^ post-index to 1-year | 0.0% |
| ED visits - post-index to 1-year | 0.0% |
| Admissions – post-index to 1-year | 0.0% |
| SNF days – post-index to 1-year | 0.0% |
| Hospice - post-index to 1-year | 0.0% |
| Mortality to one year | 0.0% |

**eTable 2.** Comparison of patients included versus excluded from the cohort of older adults transported by ambulance after a fall.

| **Demographics:** | Study cohort, including deaths  (n = 3,159) | | Excluded patients  (n = 7,469) | |
| --- | --- | --- | --- | --- |
| Age in years – median (IQR) | 84 | (77-89) | 84 | (76-89) |
| Women | 2,183 | (70.3%) | 5,151 | (70.2%) |
| **Prehospital measures:** |  | |  | |
| GCS ≤ 8 | 13 | (0.6%) | 32 | (0.6%) |
| GCS 9 – 12 | 47 | (2.1%) | 140 | (2.7%) |
| GCS 13 – 15 | 2,202 | (97.4%) | 5,090 | (96.7%) |
| SBP ≤ 100 mmHg | 132 | (4.5%) | 295 | (4.3%) |
| Heart rate ≤ 60 or ≥ 120 | 307 | (10.4%) | 717 | (10.3%) |
| Respiratory rate ≤ 10 or ≥ 28 | 42 | (1.5%) | 128 | (1.9%) |
| Receiving hospital - Level I/II trauma center | 450 | (14.3%) | 1,076 | (14.5%) |
| **Health outcome:** |  | |  | |
| 1-year mortality (%) | 665 | (21.1%) | 1,659 | (22.2%) |

**eTable 3.** Diagnoses included in the “respiratory diagnosis” variable from the index visit among older adults transported by ambulance after a fall (n = 705 patients).

1. All respiratory diagnoses, grouped by type (1,306 respiratory diagnoses in 705 patients).

|  | **Diagnoses** | **ICD9-CM codes** | **n** | **(% of 1,306 respiratory diagnosis codes)** |
| --- | --- | --- | --- | --- |
| COPD | Chronic airway obstruction; emphysema | 496, 492 | 279 | (21.4%) |
| Acute respiratory failure | Acute respiratory failure; pulmonary collapse; acute and chronic respiratory failure | 518.81, 518.0, 518.84 | 213 | (16.3%) |
| Pleurisy | Pleurisy | 511 | 141 | (10.8%) |
| Other pulmonary diseases | Other diseases of the lung, not elsewhere classified; Other diseases of respiratory system | 518.89, 519 | 124 | (9.5%) |
| Pneumonia | Pneumonia, organism unspecified; Other bacterial pneumonia; Pneumococcal pneumonia; bronchopneumonia, organism unspecified; viral pneumonia, influenza | 486, 482, 481, 485, 480, 487 | 115 | (8.8%) |
| Asthma | Asthma | 493 | 101 | (7.7%) |
| Upper respiratory diseases | Acute sinusitis; chronic sinusitis; allergic rhinitis; other diseases of upper respiratory tract; acute upper respiratory infections of multiple or unspecified sites; acute nasopharyngitis; acute pharyngitis | 491, 473, 477, 478, 465, 460, 462 | 60 | (4.6%) |
| Aspiration pneumonitis | Pneumonitis due to solids and liquids | 507 | 52 | (4.0%) |
| Chronic bronchitis | Chronic bronchitis | 491 | 42 | (3.2%) |
| Pulmonary insufficiency | Pulmonary insufficiency following trauma and surgery; other pulmonary insufficiency, not elsewhere classified | 518.5, 518.82 | 35 | (2.7%) |
| Pulmonary congestion | Pulmonary congestion and hypostasis | 514 | 34 | (2.6%) |
| Pulmonary fibrosis | Post inflammatory pulmonary fibrosis | 515 | 31 | (2.4%) |
| Chronic respiratory failure | Chronic respiratory failure | 518.83 | 19 | (1.5%) |
| Acute bronchitis | Acute bronchitis and bronchiolitis; bronchitis, not specified as acute or chronic | 466, 490 | 14 | (1.1%) |
| Pneumothorax | Pneumothorax and air leak | 512 | 13 | (1.0%) |
| Bronchiectasis | Bronchiectasis | 494 | 11 | (<1%) |
| Pulmonary eosinophilia | Pulmonary eosinophilia | 518.3 | 9 | (<1%) |
| Other alveolar disease | Other alveolar and parietoalveolar pneumonopathy | 516 | 7 | (<1%) |
| Asbestosis | Asbestosis | 501 | 2 | (<1%) |
| Lung abscess | Abscess of lung and mediastinum | 513 | 2 | (<1%) |
| Other | Respiratory conditions due to other and unspecified external agents; lung involvement in conditions classified elsewhere | 508, 517 | 2 | (<1%) |

*COPD = chronic obstructive pulmonary disease.

1. Common patient-level combinations of respiratory diagnoses (n = 705).

| **Per-patient combinations of respiratory diagnoses** | **n** | **(% of 705 patients with a respiratory diagnosis)** | **Cumulative % of 705 patients with a respiratory diagnosis** |
| --- | --- | --- | --- |
| 1. COPD | 147 | (20.9%) | 20.9% |
| 1. Asthma | 56 | (7.9%) | 28.8% |
| 1. Acute respiratory failure | 44 | (6.2%) | 35.0% |
| 1. Other pulmonary diseases | 41 | (5.8%) | 40.8% |
| 1. Pleurisy | 27 | (3.8%) | 44.6% |
| 1. Pneumonia | 18 | (2.6%) | 47.2% |
| 1. COPD; asthma | 13 | (1.8%) | 49.0% |
| 1. Pulmonary congestion | 12 | (1.7%) | 50.7% |
| 1. Acute respiratory failure; pleurisy | 11 | (1.6%) | 52.3% |
| 1. Pulmonary fibrosis | 10 | (1.4%) | 53.7% |
| 1. COPD; pneumonia | 10 | (1.4%) | 55.1% |
| 1. Pneumonia; pleurisy | 9 | (1.3%) | 56.4% |
| 1. COPD; other pulmonary diseases | 8 | (1.1%) | 57.5% |
| 1. COPD; chronic bronchitis; upper respiratory disease | 8 | (1.1%) | 58.6% |
| 1. Acute respiratory failure; other pulmonary diseases | 8 | (1.1%) | 59.7% |

*There were 201 different unique combinations of respiratory diagnoses among 705 patients. The table shows the 15 most common patient-level diagnoses and combinations of diagnoses. The additional 187 unique combinations of respiratory diagnoses each had ≤ 6 patients (<1% of patients with respiratory diagnoses).
